# CXCL Gene Clusters Regulated by Enhancer‐Mediated DNA Looping Alteration in Pancreatic Cancer Cells

**DOI:** 10.1111/jcmm.70538

**Published:** 2025-04-07

**Authors:** Yifen Shen, Yanping Hu, Hua Li, Genhai Shen, Yihang Shen, Zheng Wang

**Affiliations:** ^1^ Department of Hepatobiliary Surgery The First Affiliated Hospital of Xi'an Jiaotong University, Pancreas Center of Xi'an Jiaotong University, Xi'an Jiaotong University Xi'an Shaanxi China; ^2^ Central Laboratory Suzhou Ninth People's Hospital Suzhou Jiangsu China; ^3^ Department of Molecular Pathology The Affiliated Cancer Hospital of Zhengzhou University, Henan Cancer Hospital Zhengzhou Henan China; ^4^ Jiangsu Province Engineering Research Center of Molecular Target Therapy and Companion Diagnostics in Oncology Suzhou Vocational Health College Suzhou Jiangsu China; ^5^ Department of General Surgery Suzhou Ninth People's Hospital Suzhou Jiangsu China

**Keywords:** chromatin architecture, CXCL gene cluster, DNA loop, enhancer, pancreatic cancer

## Abstract

Pancreatic cancer is one of the deadliest cancers. Chemokines affect the progression of pancreatic cancer through various mechanisms. Most of the CXC chemokine genes, CC chemokine genes and CX3C chemokine genes are clustered together within a very short region of chromatin. Transcription activity of gene clusters is usually influenced by the chromatin architecture and spatial organisation. Nevertheless, the chromatin‐mediated regulatory mechanism on transcription of chemokine gene clusters has never been studied in pancreatic cancer. Herein, we determined that the expression of C‐X‐C motif chemokine ligand 8 (*CXCL8*), *CXCL6*, *CXCL4L1*, *CXCL1*, *CXCL4*, *CXCL7*, *CXCL5*, *CXCL3* and *CXCL2* was up‐regulated, whereas *CXCL9*, *CXCL10* and *CXCL11* were down‐regulated in pancreatic cancer cells compared with normal duct epithelial cells and further uncovered that four enhancer elements showed robust interaction to form DNA looping containing the up‐regulated eight *CXCL* genes, whereas the other enhancer controlled *CXCL9*, *CXCL10* and *CXCL11* to form another DNA loop. Furthermore, after these enhancers were respectively destroyed by CRISPR‐Cas9, we observed that the interaction with other enhancers was weakened as well as the expression of *CXCL* gene clusters and the tumour malignancy of pancreatic cancer cells was significantly changed. Taken together, our research exhibits the regulatory mechanism on transcription of *CXCL* gene clusters via enhance‐dependent DNA looping alteration in pancreatic cancer cells.

## 1 Introduction

Pancreatic cancer is one of the most aggressive and lethal malignancies worldwide. Despite its relatively lower incidence compared to other cancers, it has a disproportionately high mortality rate with the 5‐year survival rate of less than 10% [1]. Due to the lack of early symptoms and effective screening methods, most cases are diagnosed at advanced or metastatic stages, when surgical intervention is no longer an option. The median survival time for advanced pancreatic cancer is only a few months, even with treatment [2]. The presence of complicated biological characteristics, diverse tumour microenvironments as well as genetic mutations make the treatment approaches of pancreatic cancer extremely challenging. Pancreatic cancer exhibits strong resistance to chemotherapy and radiation, which reduces treatment efficacy even with the latest targeted therapies and immunotherapies, the effectiveness is often limited. Therefore, pancreatic cancer is dubbed the ‘king of cancers’ driving urgent demand for research and improved treatment strategies [3].

Various mechanisms influence pancreatic cancer development and progression by chemokines, including promoting cell migration, immune evasion and metastasis [4]. The CXCL12‐CXCR4 axis, often overexpressed in pancreatic cancer and guiding cancer cells to distant organs, such as the liver and lungs, plays a crucial role in the migration and metastasis of pancreatic cancer cells [5, 6]. CCL2 establishes an immunosuppressive tumour microenvironment by attracting immune cells like tumour‐associated macrophages (TAMs). TAMs can promote cancer cell survival and growth [7], thereby enabling the tumour to evade immune detection [8]. In addition, the process of angiogenesis, the formation of new blood vessels, provides nutrients to the growing tumour through chemokines like CXCL8 (IL‐8) [9]. CXCL8 can also enhance the invasive properties of pancreatic cancer cells, contributing to tumour progression. Certain chemokines contribute to the resistance of pancreatic cancer to chemotherapy. For instance, CCL5 interacts with the CCR5 receptor on cancer cells, making them less responsive to treatment [10].

Notably, CXC chemokine family (Chr4q13‐21), CC chemokine family (Chr17q11.2‐q21.1), CX3C family (Chr16q13), C family members *XCL1* and *XCL2* (Chr1q23) are all clustered together within a very short region of chromatin. Chemokine receptors, such as *CCR1*, *CCR2*, *CCR3*, *CCR5*, *CCR9*, *CXCR6*, *XCR1* and *CX3CR1*, are also gathered in Chr3p21. A gene cluster usually refers to a group of genes that are located near each other on a chromosome. These genes share similarities in sequence as well as encode proteins with related functions, typically evolving from a common ancestral gene and arising from duplication and divergence, but they have developed functional differences in the metabolic pathway or signalling process over time. However, these genes are still frequently regulated by the same mechanisms, such as sharing identical promoters, enhancers or other regulatory elements to allow them to be activated or repressed together under certain conditions [11]. In the context of chromatin architecture, these gene clusters can be influenced by the spatial organisation and dynamic changes in chromatin that determine the accessibility of genes for transcription [12]. Studying gene clusters helps people understand how multiple elements collaborate to control complex biological processes. For example, mutations in the β‐globin gene cluster or upstream enhancers (DNase hypersensitive sites) are linked to thalassaemia [13]. Research into gene clusters can help unravel the intricate coordination of gene expression, reveal how groups of genes work together to perform essential biological functions and aid in disease diagnosis and therapy. Nevertheless, the chromatin architecture‐mediated regulatory mechanism on the transcription of chemokine gene clusters has never been studied in pancreatic cancer.

Here, we use the *CXCL* gene cluster as an example to study the relationship and impact between epigenetic regulation and transcriptional control of the *CXCL* gene cluster within a 400‐kb region on chromosome 4 in pancreatic cancer. Our research suggests that simultaneous up‐regulation and down‐regulation of multiple chemokines in pancreatic cancer show promising potential for therapeutic transformation. This approach can also be extended to other tumours, providing a new framework for studying the transcriptional regulation of numerous tumour‐suppressing or tumour‐promoting factors.

## 2 Materials and Methods

### 2.1 Bioinformatic Analysis

Public datasets of ChIP‐seq and CUT&TAG on pancreatic cancer cells were used in this study. Accession numbers in GEO DataSets in NCBI were listed below. GSM4490504, GSM4490505, GSM4490506, GSM4490526, GSM4490527, GSM4490528, GSM5112038, GSM5112039, GSM5112046, GSM5112047, GSM5112398, GSM5112399, GSM5330474, GSM5330475, GSM5330481, GSM5330482, GSM5330483, GSM5330484, GSM5330881, GSM5330882, GSM5330883, GSM5331141, GSM5331142, GSM5331143, GSM6940293, GSM6940294, GSM6940297, GSM7610454, GSM7610458, GSM7610461, GSM7884636, GSM7884637, GSM7884638, GSM7884639 and GSM7884640.

### 2.2 Cell Culture

PANC‐1 (Cat. no. CRL‐1469), MIA PaCa‐2 (Cat. no. CRM‐CRL‐1420) and HPNE (Cat. no. CRL‐4023) cells were obtained from ATCC (USA). H6C7 cells were obtained from Kerafast (USA). PANC‐1, MIA PaCa‐2 and H6C7 cells were cultured in Dulbecco's Modified Eagle's Medium (DMEM) with 10% fetal bovine serum (FBS) (Thermo Fisher Scientific, USA). HPNE cells were cultured in 75% DMEM and 25% M3 Base medium (Incell, USA) supplemented with 2 mM L‐glutamine and 1.5 g/L sodium bicarbonate.

### 2.3 CRISPR‐CAS9‐Driven Genetic Editing

Five enhancer regions around the *CXCL* gene cluster were picked up based on the database of EnhancerAtlas (http://www.enhanceratlas.org/downloadv2.php) [14] and ENdb (http://www.licpathway.net/ENdb) [15] (Table S1). Guide DNA (Table S1) was designed by ‘CRISPOR’ engine from Zhang Lab (http://crispor.gi.ucsc.edu/) and inserted after the U6 promoter of LentiCRISPR v2‐EGFP (Addgene, USA). Plasmids were transfected into cells using Lipofectamine 3000. After 72 h, GFP‐positive cells were selected by BD FACSARIA III (BD Biosciences, USA) and subcultured into a 15‐cm dish with a density of 1 × 10^5^. After 2‐week culture, single‐cell clones with positive GFP were picked up by cloning rings (Millipore, USA) and passaged and cryopreserved. PCR and Sanger sequencing were used to identify the efficiency of knockout.

### 2.4 Quantitative Polymerase Chain Reaction (qPCR)

Template cDNA generated from mRNA or purified genomic DNA was used for qPCR. Aurum total RNA Mini Kit (Bio‐Rad) was used to isolate RNA. iTaq Universal SYBR Green One‐Step Kit (Bio‐Rad) was used to synthesise cDNA and prepare the PCR system. PCR conditions were not significantly modified, as suggested in the specification, 95°C 2 min for initial denaturation; 95°C 10 s, 60°C 30 s, 72°C 30 s for 40 cycles. $2^{-∆∆C_{T}}$ method was used to assess the relative copy number of the targets in different samples compared with the control. Primers sequence was listed in Table S1.

### 2.5 Chromatin Immunoprecipitation (ChIP)‐qPCR

Final concentration of 2% paraformaldehyde was added into medium to fix the cells at least 1 × 10^8^ for 10 min at room temperature; then, 0.125 M glycine was added to quench the crosslinking for 15 min. Cells were scraped off from the dish using a cell scraper, centrifuged under 3000 rpm for 15 min and washed with cold PBS twice. Cell pellets were resuspended in 1 mL cold Pierce IP buffer (Thermo Fisher Scientific) with 1× Protease Inhibitor Cocktail (MedChemExpress, China) and remained on ice for 30 min with occasional vortexing. After centrifugation under 15,000 rpm for 15 min, the supernatant was transferred into another Eppendorf tube. The supernatant was treated with Bioruptor UCD200 Sonication (Diagenode, Belgium) to shear the genomic DNA. Ten microlitre was stored as input. Nine hundred and fifty microlitre was divided into two pieces of 450 μL and supplemented to 1 mL and incubated with 1 μg antibodies against H3K4me1 (Cat. no. 5326, CST, USA) or H3K27ac (Cat. no. 8173, CST), respectively overnight. The next day, 20 μL Pierce Protein A/G magnetic beads (Thermo Fisher Scientific) were added to the IP system. After another 2‐h incubation, the beads were washed with LiCl wash buffer (0.25 M LiCl, 1% IGEPAL CA‐630, 1% sodium deoxycholate, 1 mM EDTA 10 mM Tris‐Hcl, pH 8.0), high salt wash buffer (500 mM NaCl, 0.1% SDS, 1% IGEPAL CA‐630, 2 mM EDTA, 20 mM Tris‐Cl, pH 8.0) and low salt wash buffer (150 mM NaCl, 0.1% SDS, 1% IGEPAL CA‐630, 2 mM EDTA, 20 mM Tris‐Cl, pH 8.0) three times each in order. DNA on beads and in input was isolated using the PureLink Genomic DNA Mini Kit (Thermo Fisher Scientific). qPCR was performed to detect the abundance of target regions.

### 2.6 Chromosome Conformation Capture (3C)‐qPCR

Final concentration of 2% paraformaldehyde was added into medium to fix the cells at least 1 × 10^8^ for 10 min at room temperature; then, 0.125 M glycine was added to quench the crosslinking for 15 min. Cells were scraped off from the dish using a cell scraper, centrifuged under 3000 rpm for 15 min and washed with cold PBS twice. Cell pellets were resuspended in 1 mL cold Pierce IP buffer (Thermo Fisher Scientific) with 1× Protease Inhibitor Cocktail (MedChemExpress, China) and stayed on ice for 30 min with occasional vortexing. The first round of centrifugation under 1000 rpm for 15 min was performed to obtain nuclei in the supernatant. The second round of centrifugation under 15,000 rpm for 30 min was performed, and the supernatant was removed. The nuclear precipitation was washed with 1 mL NEBuffer r2.1 (NEB, USA), and 300 μL NEBuffer r2.1 and 3 μL 10% SDS were added at 65°C for 10 min, then 16 μL 20% Triton X‐100 was added at 37°C for 1 h, then 500 U Mse I (NEB) for digestion at 37°C overnight. The next day, 30 μL 10% SDS was added at 65°C for 20 min, and 350 μL 20% Triton X‐100 was added; then, water was added to 6 mL at 37°C for 1 h, then 700 μL ligase buffer, 20 μL T4 Ligase, 70 μL BSA and water were added to 7 mL at 16°C overnight. DNA was purified using the PureLink Genomic DNA Mini Kit as template for qPCR. DNase I hypersensitive site 2 in the locus control region of human β‐globin was used as a positive control as previously described [16, 17]. Two random genomic regions in different chromosomes were used as a negative control.

### 2.7 CCK‐8 Assay

Cells were seeded into a 96‐well plate at a density of 1 × 10^3^ per well. Plates were incubated at 37°C in a 5% CO_2_ incubator overnight to allow cells to adhere and grow. Ten microlitre of CCK‐8 reagent (MCE, China) were directly added into each well. After another 4‐h culture, absorbance at 450 nm was measured using a Multiskan FC microplate reader (Thermo Fisher Scientific) to evaluate the number of viable cells. The absorbance readings by subtracting the background were used to normalise those from different groups of cells before analysis.

### 2.8 Transwell Assay

Cells were seeded into the upper chamber of the transwell insert (Corning, USA) in serum‐free medium. The lower chamber contained medium with 10% FBS, which induced cell movement from the upper to the lower chamber. Plates were incubated at 37°C in a 5% CO_2_ incubator for 48 h. After the incubation period, medium was carefully removed, cells that remained on the upper side of the membrane were gently removed using a cotton swab, and the migrated cells were fixed by 4% paraformaldehyde, then stained by 1 mL crystal violet (Cat. no. C0121, Beyotime, China) per well for 30 min at room temperature. After washing by PBS twice as well as air drying, the migrated cells on the bottom side of the membrane were retained for analysis. The migrated cells were visualised under a microscope, and the number of cells in five fields of view was counted and analysed for quantification.

### 2.9 Annexin V/PI for Cell Apoptosis Assay

The collected cells were suspended once with pre‐cooled 1× PBS, centrifuged at 300 *g* for 5 min, the supernatant was discarded to collect the cells, 300 μL of 1× binding buffer was added to resuspend the cells, and the cell concentration was adjusted to 1 × 10^6^/mL. The 100 μL cell suspension was filtered through 200‐mesh membranes and added to the flow tube and gently mixed with 5 μL Annexin V‐alexa Fluor 488 (Invitrogen, USA) and 10 μL PI (Sigma, USA). For the care, add the above two kinds of dyeing solution respectively. After incubation at room temperature for 15 min, 400 μL 1× binding buffer was added and placed in an ice bath for detection by LSRFortessa X‐20 (BD Biosciences, USA). The selected channel is excitation wavelength of 488 nm and acquisition band 530/30 for Annexin V as well as excitation wavelength of 560 nm and acquisition band 610/20 for PI. Annexin V was positive for early apoptotic cells, PI positive for necrotic cells, Annexin V and PI positive for late apoptotic cells and Annexin V and PI negative for living cells. Early and late apoptotic cells were included in the statistical analysis.

### 2.10 Statistical Analysis

The experiment was repeated three times for each sample. The average and standard errors of the calculated data were used in the preparation of the chart. One‐way ANOVA was used to define the statistical significance (*p* value less than 0.05).

## 3 Results

### 3.1 DNA Loop Conformation of CXCL Gene Cluster in Pancreatic Ductal Adenocarcinoma

**FIGURE 1:**
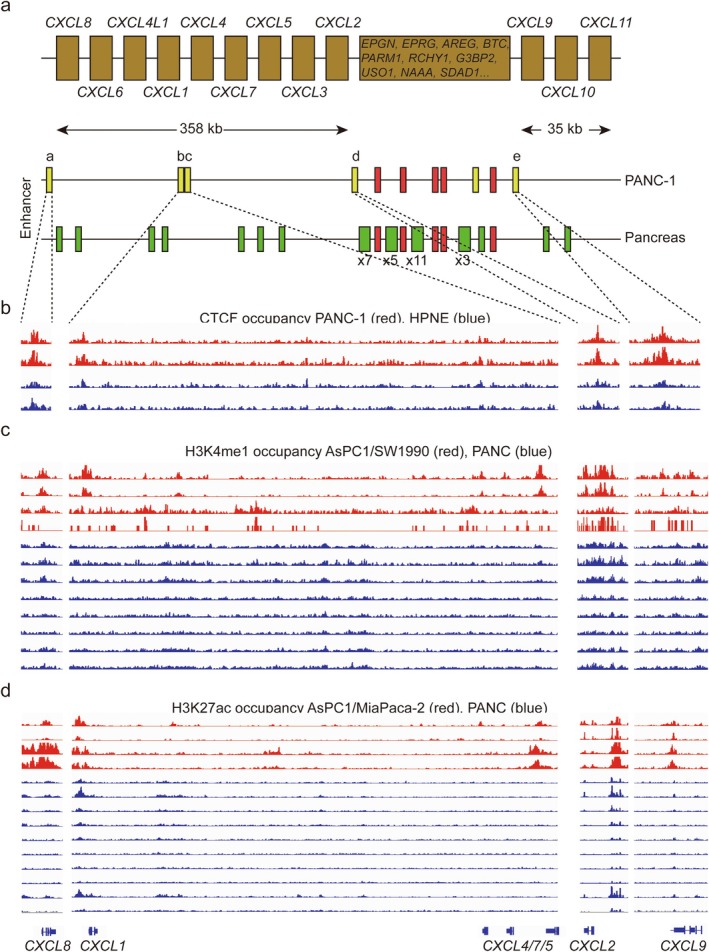
Epigenetic information of *CXCL* gene clusters in pancreatic cancer cells. (a) Schematic representation of the enhancer location in *CXCL* gene clusters in PANC‐1 cells and pancreas based on EnhancerAtlas Database. Yellow boxes marked by ‘a’, ‘b’, ‘c’, ‘d’, ‘e’ represent five pancreatic cancer specific enhancer elements which are focused on in this article. Red boxes represent the mutual enhancer elements in pancreatic cancer and normal pancreas. Green boxes represent the enhancer elements only appear in normal pancreas. Due to the scale and size, it is more intuitive to combine multiple boxes together, such as ‘×3’ means there are three enhancers within one box, and so on. (b) IGV shows the CTCF occupancies on enhancers ‘a’ to ‘e’ of CXCL gene cluster in pancreatic cancer cells and normal pancreatic tissues or cells by public database. Red peaks are from CTCF ChIP‐seq data of pancreas ductal adenocarcinoma PANC‐1 cells (Accession nos. GSM4490526, GSM4490527). Blue peaks are from ChIP‐seq data of normal ductal HPNE cells assigned GSM4490504, GSM4490505. CTCF peaks in IP groups have been normalised by input datasets. (c) H3K4me1 enrichments on enhancers of CXCL gene cluster. Red peaks are from pancreas ductal adenocarcinoma AsPC1 cells without (GSM7884639) and with gemcitabine resistance (GSM7884640), SW1990 by ChIP‐seq (GSM7610454) and CUT&TAG (GSM7610461). Blue ones are from eight replications of three human normal pancreas (PANC) (GSM5331141, GSM5331142, GSM5331143, GSM5330881, GSM5330882, GSM5330883, GSM5330474, GSM5330475). (d) H3K27ac enrichments on enhancers of CXCL gene cluster. Red peaks are from AsPC1 cells without (GSM7884637) and with gemcitabine resistance (GSM7884638), MIAPaca‐2 (GSM6940293, GSM6940294). Blue ones are from 10 replications of four human normal pancreas (PANC) (GSM5330481, GSM5330482, GSM5330483, GSM5330484, GSM5112398, GSM5112399, GSM5112046, GSM5112047, GSM5112038, GSM5112039). The display of CXCL labels at the bottom help intuitively see the real distance between CXCLs and enhancers on the genome. Their location is provided by IGV software. (b–d) Share the same label of these CXCLs at the bottom.

The human *CXCL* gene family consists of 17 members, 12 of which are densely clustered in two regions of chromosome 4, with lengths of 358 and 35 kb, respectively (Figure 1a). In the perspective of 3D chromosome architecture, the transcription of nearby genes is often influenced by the same distal regulatory element. Therefore, these 12 *CXCL* genes are likely to exhibit similar transcriptional activity. By the database of EnhancerAtlas and ENdb, we noticed that there were 11 enhancers in PANC‐1 cells, whereas 41 enhancers were found in normal pancreatic tissue. In addition to the five mutual enhancers, six other tumour‐specific enhancer elements (yellow frames) were uniquely present in PANC‐1 cells, and five enhancers (termed ‘a’ to ‘e’) distributed within or nearby the *CXCL* gene cluster, which provoked our interest (Figure 1a). Public data of CTCF, H3K4me1 and H3K27ac on the *CXCL* gene cluster in pancreatic cancer were also shown. As the essential role in enhancer–promoter interactions [18], CTCF was highly enriched on enhancer ‘a’ (log_2_FC = 3.48, *p* = 5.47 × 10^−6^), ‘d’ (log_2_FC = 5.12, *p* = 3.26 × 10^−8^) and ‘e’ (log_2_FC = 5.54, *p* = 1.07 × 10^−8^), which seems to isolate this gene cluster from the outside, but there was no significance within ‘b’ and ‘c’ in PANC‐1 cells compared to HPNE cells (Figure 1b). Furthermore, we also found that H3K4me1 and H3K27ac, as the hallmarks of enhancer, were remarkably elevated in these enhancer elements (H3K4me1: ‘a’, log_2_FC = 1.38, *p* = 2.7 × 10^−3^, ‘d’, log_2_FC = 3.15, *p* = 3.18 × 10^−6^, ‘e’, log_2_FC = 1.62, *p* = 3.07 × 10^−3^; H3K27ac: ‘a’, log_2_FC = 7.48, *p* = 5.47 × 10^−10^, ‘d’, log_2_FC = 4.03, *p* = 8.1 × 10^−7^, ‘e’, log_2_FC = 3.27, *p* = 4.97 × 10^−6^) in pancreatic cancer compared to normal pancreatic tissues or cells (Figure 1c,d).

**FIGURE 2:**
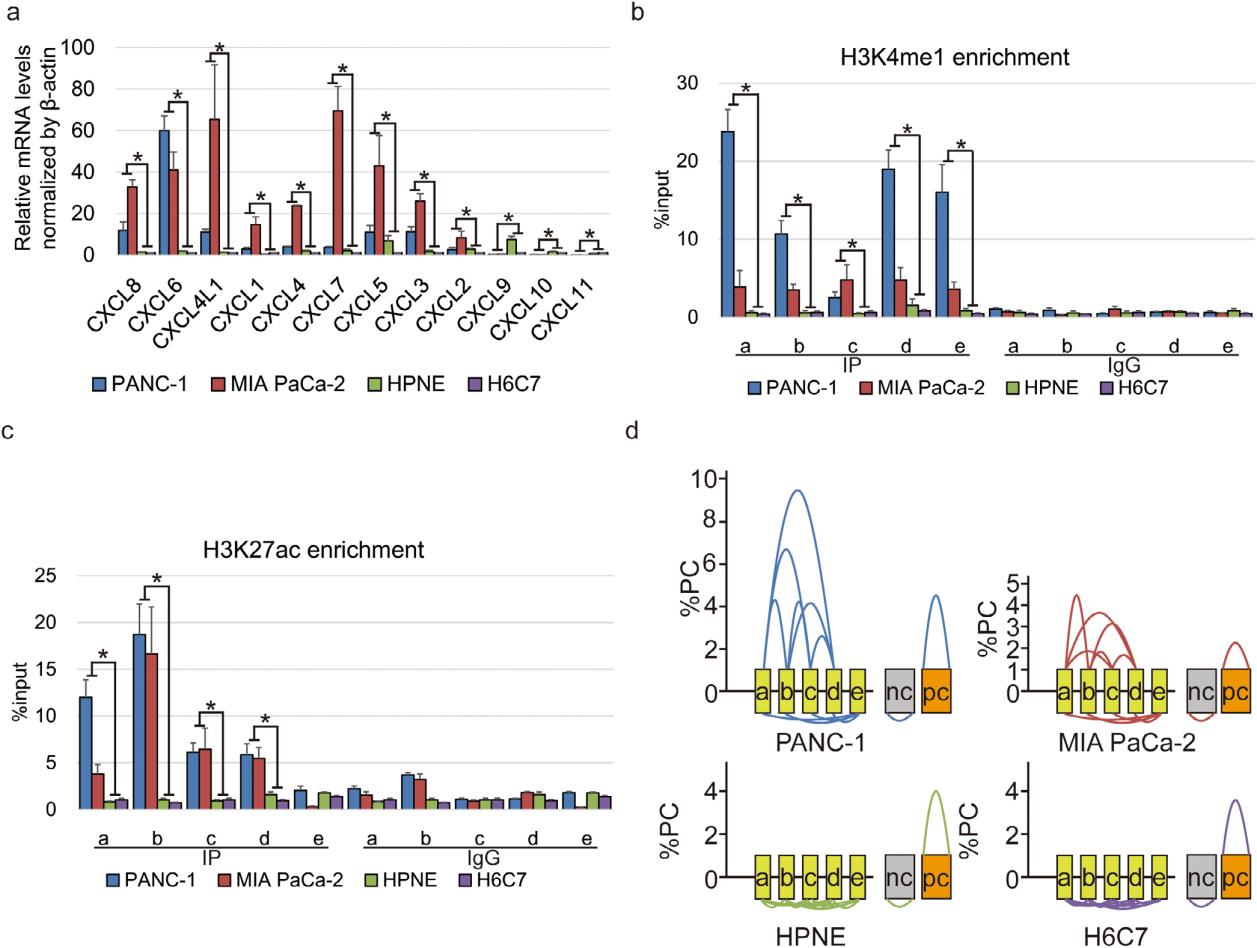
Chromatin architecture of *CXCL* gene clusters in pancreatic cancer cells. (a) Histogram indicates the relative mRNA levels of *CXCL* gene clusters in pancreas ductal adenocarcinoma PANC‐1 and MIA PaCa‐2 cell lines and normal ductal HPNE and H6C7 cell lines. ‘*’ Represents the comparison between three biological replications of cancer cells and three biological replications of normal cells with *p* value less than 0.05 by one‐way ANOVA. (b, c) Histogram indicates the enrichment of H3K4me1 (b) or H3K27ac (c) on enhancer ‘a’ to ‘e’ in pancreas ductal adenocarcinoma and normal ductal cells. ‘*’ Represents the comparison between three biological replications of cancer cells and three biological replications of normal cells with *p* value less than 0.05 by one‐way ANOVA. (d) Diagram indicates the interaction among enhancer ‘a’ to ‘e’ in pancreas ductal adenocarcinoma and normal ductal cells. The height from the vertex of the curve to the *X*‐axis represents the relative strength of the interaction to positive control (% PC) by three biological replications. Curves above the box indicate the statistical significance (*p* value less) by one‐way ANOVA. PC: positive control, HS2 to β‐globin promoter; NC: random genomic regions in different chromosomes; PC: DNase I hypersensitive site 2 in the locus control region of human β‐globin as a positive control.

Here, mRNA levels of 12 *CXCL* genes in two pancreas ductal adenocarcinoma PANC‐1 and MIA PaCa‐2 cell lines as well as normal ductal HPNE and H6C7 cell lines were indicated by qPCR. We found that the expression of *CXCL8*, *CXCL6*, *CXCL4L1*, *CXCL1*, *CXCL4*, *CXCL7*, *CXCL5*, *CXCL3* and *CXCL2* was up‐regulated, whereas *CXCL9*, *CXCL10* and *CXCL11* were down‐regulated in cancer cells compared to normal duct epithelial cells (Figure 2a). ChIP‐qPCR for H3K4me1 and H3K27ac was performed to verify the enhancer activity in these regions indicated by public data. We determined that H3K4me1 was positively presented in all these five regions (Figure 2b), whereas H3K27ac was positively shown in enhancers ‘a’ to ‘d’ not ‘e’ in PANC‐1 and MIA PaCa‐2 cells (Figure 2c) compared to HPNE and H6C7 cells, indicating a transcriptional silencing on enhancer ‘e’ in pancreatic cancer cells. Moreover, 3C‐qPCR assay confirmed that enhancers ‘a’ to ‘d’ displayed a robust proximal–distal interactions, but not to enhancer ‘e’ in pancreatic cancer cells (Figure 2d). These evidence indicated that DNA loop conformation was closely related to transcription of the *CXCL* gene cluster in pancreatic cancer.

### 3.2 Transcription of CXCL Gene Cluster Affected by Enhancer Destruction

**FIGURE 3:**
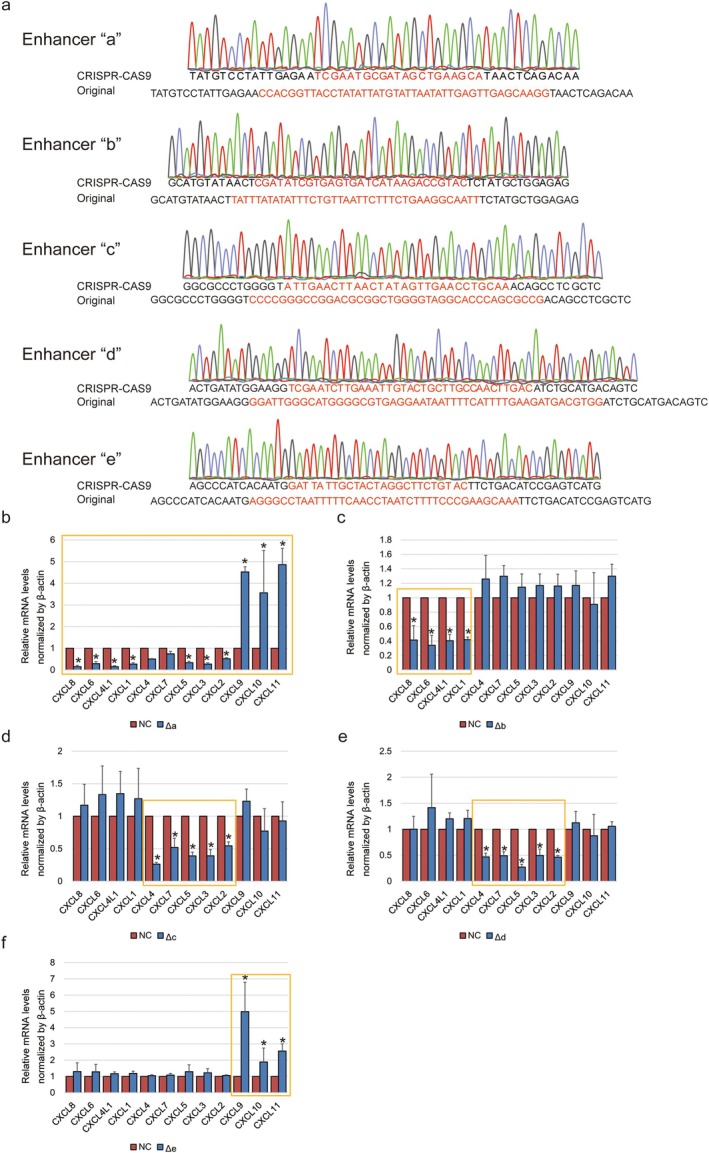
Transcription alteration of *CXCL* gene clusters affected by enhancer destruction. (a) Sanger sequencing show the enhancer ‘a’ to ‘e’ in PANC‐1 cells after being destroyed by the Crispr‐cas9 system. Sequences marked in red are new generated (above) compared to wild type one (below) in each enhancer. (b–f) Histogram indicates the relative mRNA levels of *CXCL* gene clusters in wild type PANC‐1 cells compared to the ones with enhancer destruction by three biological replications. (b) Enhancer ‘a’ destruction; (c) enhancer ‘b’ destruction; (d) enhancer ‘c’ destruction; (e) enhancer ‘d’ destruction; (f) enhancer ‘e’ destruction. NC: wild type PANC‐1; Δ: destruction. ‘*’ Represents the comparison between wild type PANC‐1 cells and the ones with enhancer destruction with *p* value less than 0.05 by one‐way ANOVA. Yellow frames highlight the significant differences.

To further study the role of DNA loop conformation in *CXCL* gene transcription, five enhancers in PANC‐1 cells were partially destroyed by CRISPR‐CAS9 (Figure 3a). Enhancer ‘a’ deletion compromised most of the expression of *CXCL* genes, but strengthened the expression of *CXCL9*, *CXCL10* and *CXCL11* in turn (Figure 3b). Enhancer ‘b’ only weakened the expression of *CXCL8*, *CXCL6*, *CXCL4L1* and *CXCL1* (Figure 3c). Enhancer ‘c’ and ‘d’ could both reduce the expression of *CXCL4*, *CXCL7*, *CXCL5*, *CXCL3* and *CXCL2* (Figure 3d,e). Enhancer ‘e’ deletion unexpectedly elevated the expression of *CXCL9*, *CXCL10* and *CXCL11* in PANC‐1 cells (Figure 3f).

### 3.3 DNA Loop Conformation Alteration Affected by Enhancer Destruction

**FIGURE 4:**
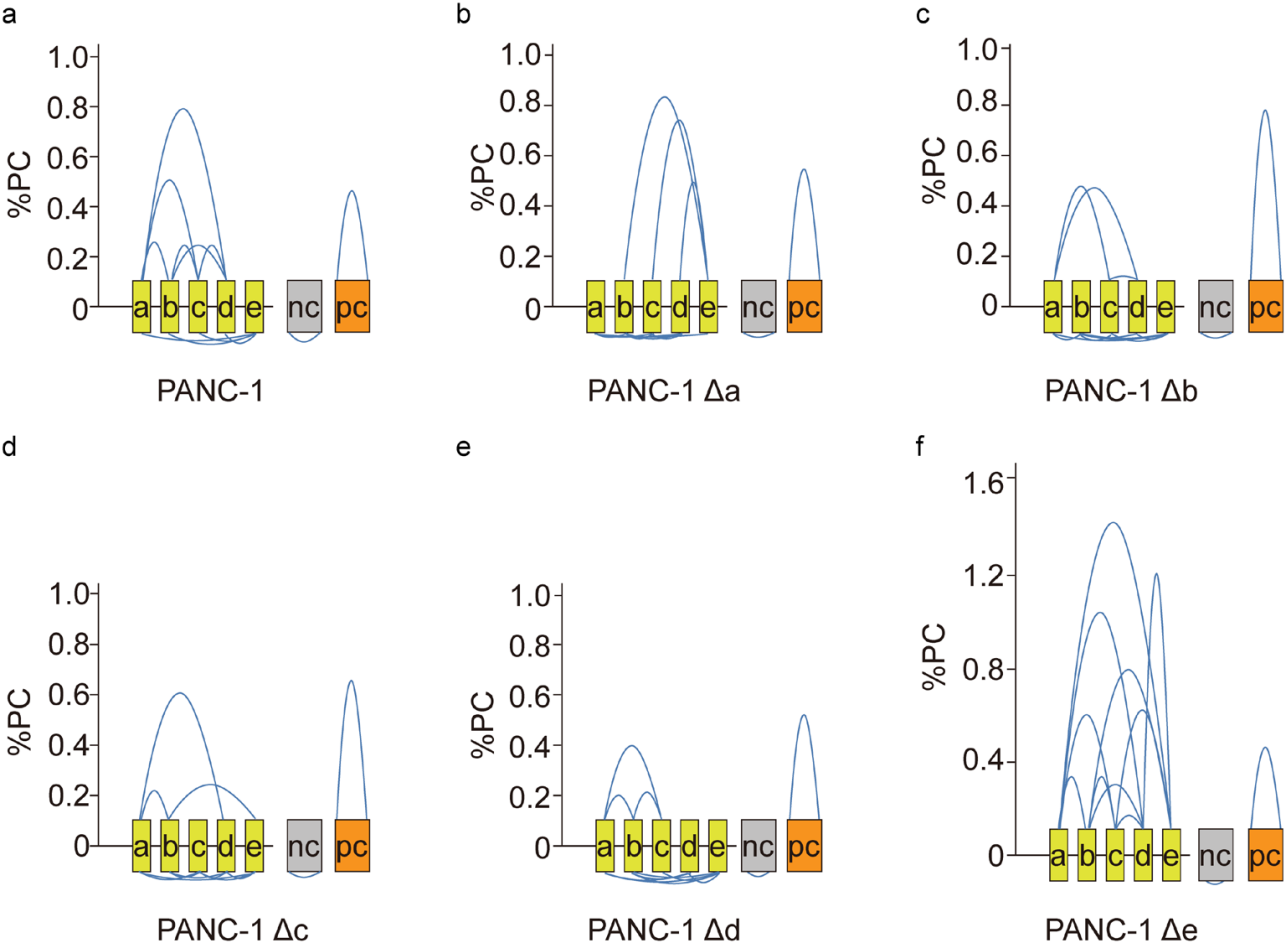
Chromatin architecture of *CXCL* gene clusters affected by enhancer destruction. (a–f) Diagram indicate the interaction among enhancer ‘a’ to ‘e’ in wild type PANC‐1 cells compared to the ones with enhancer destruction by three biological replications. (a) Wild type; (b) enhancer ‘a’ destruction; (c) enhancer ‘b’ destruction; (d) enhancer ‘c’ destruction; (e) enhancer ‘d’ destruction; (f) enhancer ‘e’ destruction.

Consistently, 3C‐qPCR validated that enhancer ‘a’ deletion impaired the interactions among enhancers ‘a’, ‘b’, ‘c’ and ‘d’, meanwhile facilitating the interactions of enhancer ‘b’, ‘c’, ‘d’ with enhancer ‘e’ (Figure 4a). Moreover, corruption of enhancer ‘b’, ‘c’ or ‘d’ only demolished their own interactions with other enhancers (Figure 4b–d). Interestingly, alteration of enhancer ‘e’ compared to the alteration of enhancer ‘b’, ‘c’ or ‘d’ could result in the robust interaction among enhancer ‘e’ with enhancer ‘a’ to ‘d’ (Figure 4e). Using the CRISPR‐Cas9‐driven knockout method, we determined that the DNA loop conformation indeed settled on the transcription of the *CXCL* gene cluster.

### 3.4 Effects of DNA Loop Conformation on Malignancy of Pancreatic Cancer

**FIGURE 5:**
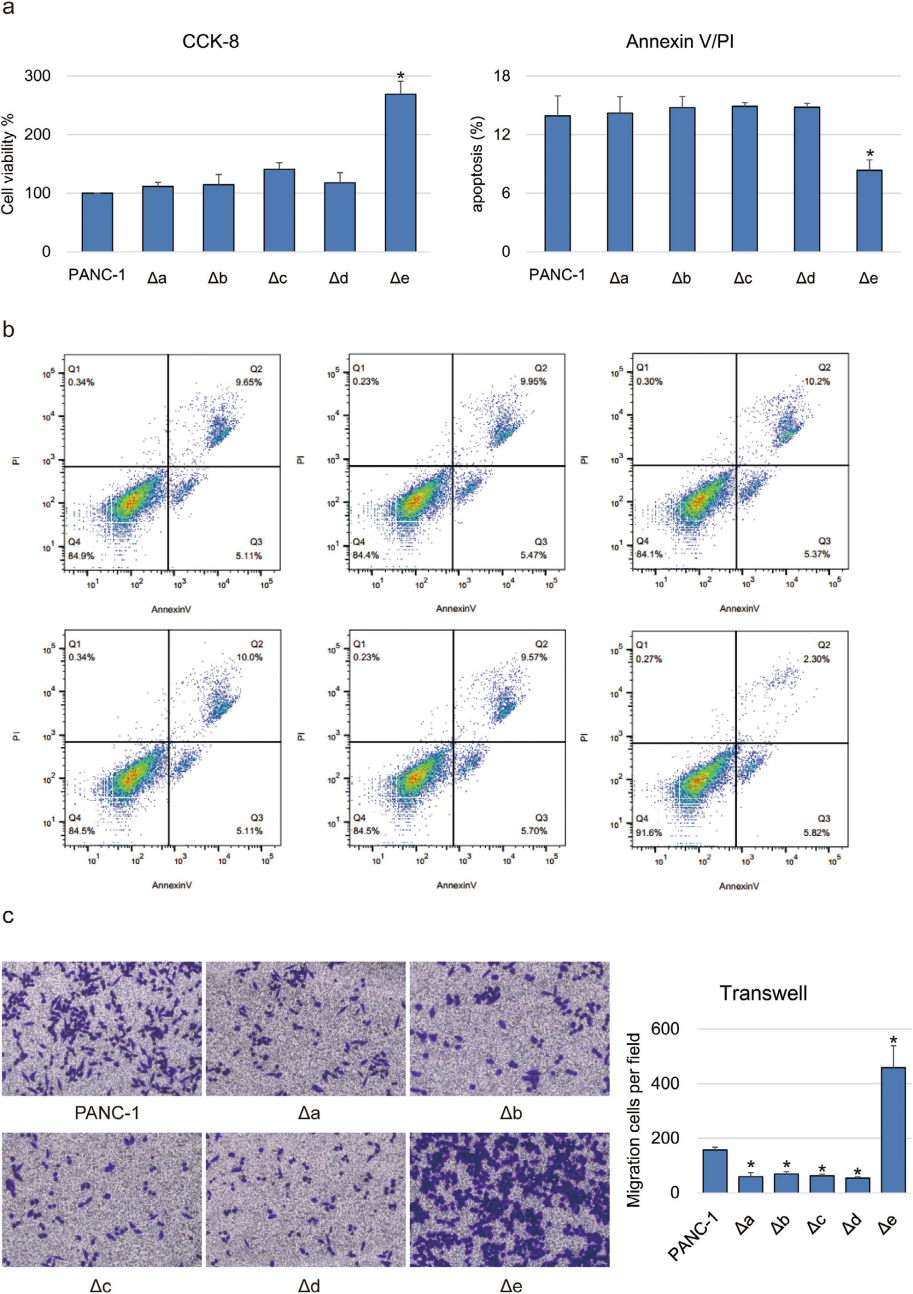
Tumour malignancy of PANC‐1 cells affected by enhancer destruction. (a) Histogram indicate cell viability of PANC‐1 with enhancer destruction by three biological replications. (b) Annexin V/PI method indicate the cell apoptosis of PANC‐1 with enhancer destruction. Annexin V positive cells (quadrant one and four) are considered as apoptotic cells. The histogram in the upper right corner is comparative analysis of apoptotic cell numbers. (c) Transwell assay indicate the cell migration of PANC‐1 with enhancer destruction. The histogram in the right side is comparative analysis of migrated cell numbers. ‘*’ Represents the comparison between three biological replications of wild type PANC‐1 cells and the ones with enhancer destruction with *p* value less than 0.05 by one‐way ANOVA.

Due to DNA looping leading to remarkable transcription alteration of multiple genes not only *CXCL* genes, we examined the impact of this DNA looping mechanism on tumour malignancy of pancreatic cancer by in vitro assay for cell growth, migration and apoptosis. Deletion of enhancer ‘a’ to ‘d’ failed to affect cell proliferation (Figure 5a) nor apoptosis (Figure 5b), but obviously compromised the ability of cell migration (Figure 5c), with the enhancer ‘a’ displaying the most significant effect. On the other hand, deletion of enhancer ‘e’ could strengthen the cell multiplication and migration, as well as reduce the apoptosis. Our observations indicated the different effects of enhancers on tumour malignancy of pancreatic cancer.

## 4 Discussion

The study of the *CXCL* gene cluster in pancreatic cancer has shown that these genes play crucial roles in tumour initiation, progression and regulation of the tumour microenvironment. Nevertheless, some members of the *CXCL* family exhibit complex roles in pancreatic cancer, potentially acting differently depending on the environment or stage of tumour development. The *CXCL* family plays both tumour‐promoting (pro‐tumourigenic) and tumour‐suppressing (anti‐tumourigenic) roles depending on the specific chemokine, its receptor and the tumour microenvironment. Notable pro‐tumour *CXCL* members include *CXCL1*, *CXCL2*, *CXCL5* and *CXCL8*, which influence cell migration, angiogenesis, inflammatory responses and immune regulation [19, 20, 21, 22]. Other *CXCL* family members, particularly those involved in immune surveillance and activation, contribute to inhibiting tumour growth by enhancing anti‐tumour immune responses and limiting angiogenesis, such as *CXCL9*, *CXCL10* and *CXCL11* [23]. Another two *CXCL* genes, *CXCL4* and *CXCL12*, have dual roles that they promote or inhibit cancer depending on the context [24, 25]. This indicates that although CXCL genes locate within the same gene cluster, their protein functions have diverged significantly, exhibiting completely opposite effects, at least in terms of anti‐tumour activity.

Now, our findings have suggested that the transcription of *CXCL9*, *CXCL10* and *CXCL11* in pancreatic cancer cells is the opposite of that of the other eight *CXCL* genes. Coincidentally, *CXCL9*, *CXCL10* and *CXCL11* are separated from the other eight *CXCL* gene clusters by more than 10 genes, although on a larger scale they are located very close to each other on chromosome 4. The difference in the function of cancer promotion and suppression, as well as the different positioning of the genome, so perfectly fit; this ‘coincidence’ undoubtedly shows the beauty of life science [26]. One of the most important innovations of this study is the discovery of different transcriptionally active regions in the *CXCL* gene clusters, which are driven by the related enhancers. We can notice that enhancers from ‘a’ to ‘d’ form a relatively independent DNA loop showing more robust interaction among each other beyond ‘e’. Eight *CXCL* genes included within this compartment display a similar epigenetic pattern and transcription activity (Figure 1). Once these enhancers are destroyed, Figures 2 and 3 show that the whole DNA loop subsequently disappears, following the altered transcription of *CXCL* genes. Undoubtedly, advances in 3D genomics and epigenetics underscore enhancer looping as a central mechanism in CXCL‐driven oncogenesis, which has never been involved in previous studies. Of course, changes in enhancer activity mediated by DNA looping represent only a small aspect of epigenetics. Many other epigenetic factors influence the regulation of CXCL expression, including DNA methylation, RNA methylation and Polycomb group (PcG) proteins [27].

Notably, in pancreatic cancer cells, enhancer ‘a’ and enhancer ‘e’ function similarly because upon these two enhancers driving, *CXCL* gene clusters form two DNA loops with completely independent transcriptional activity. The representative genes are the first eight *CXCL* genes and the last three *CXCL* genes. The difference is that enhancer ‘a’ xerts transcriptional activation downstream, while enhancer ‘e’ exerts transcriptional inhibition downstream. The destruction of enhancers ‘b’, ‘c’ and ‘d’ only affects the nearby *CXCL* gene transcription, indicating a weaker influence on gene transcription than enhancer ‘a’ and enhancer ‘e’. This is unique to pancreatic cancer cells. In normal pancreatic tissues or cells, these enhancers all disappear. Enhancer is defined as positive H3K4me1 enrichment, whereas active enhancer is defined as positive H3K27ac enrichment [28, 29]. In particular, CTCF must be crucial in regulating the enhancer–gene interactions [30]. By the analysis of public datasets, we have noticed that CTCF helps to act as a wall, separating specific genes from the outside world and helping to control the regulatory range of enhancers. Studying the enhancer formation mechanism in the *CXCL* gene cluster in pancreatic cancer is highly significant for the treatment of pancreatic cancer. The roles of CTCF, such as the CTCF binding site orientation, protein complex at CTCF binding sites, as well as the alteration of chromatin architecture and organisation by CTCF in pancreatic cancer, will also be one of the issues we need to address in the future.

In addition, there are also above 10 genes between *CXCL2* and *CXCL9*; these genes' expression will undoubtedly be affected by the enhancer deletion. The overall effects on the malignancy of pancreatic cancer cells are evaluated in Figure 4. *CXCL* genes majorly contribute to the changeable migration capability, except for proliferation and apoptosis, even if other 10 genes are up‐ or down‐regulated. Therefore, we can demonstrate that genetic editing of these enhancers can locally affect a small range of genes.

Taken together, our research exhibits the regulatory mechanism on the transcription of CXCL gene clusters via enhancer‐dependent DNA looping alteration in pancreatic cancer cells.

## Author Contributions

**Yifen Shen:** data curation (equal), formal analysis (equal), investigation (equal), methodology (equal). **Yanping Hu:** data curation (equal), formal analysis (equal), investigation (equal), methodology (equal), resources (equal), software (equal), validation (equal), visualization (equal). **Hua Li:** resources (equal), software (equal). **Genhai Shen:** resources (equal), software (equal). **Yihang Shen:** conceptualization (equal), data curation (equal), funding acquisition (equal), investigation (equal), supervision (equal), validation (equal), writing – original draft (equal). **Zheng Wang:** conceptualization (equal), project administration (equal), supervision (equal), writing – review and editing (equal).

## Conflicts of Interest

The authors declare no conflicts of interest.

## Supporting information


**Table S1.** The sequence of primers and enhancers are listed.

## Data Availability

Data will be made available on request.
