# Supplementary material for: CXCL Gene Clusters Regulated by Enhancer‐Mediated DNA Looping Alteration in Pancreatic Cancer Cells
Source: J Cell Mol Med. 2025 Apr 7;29(7):e70538. doi: 10.1111/jcmm.70538 (PMC11975504; doi:10.1111/jcmm.70538)
Supplement: Supplementary file 1 — Table S1. The sequence of primers and enhancers are listed. [file JCMM-29-e70538-s001.docx]

| Symbol name | Sequence | Tm (^o^C) |
| --- | --- | --- |
| CXCL1 | tggtagccgctggccggcgcgc  ACTATGGGGGATGCAGGATTGAGGCA | 58 |
| CXCL2 | ggcca ctgaactgcgctgccagtgcttgcag  CCATTTTTCAGCATCTTTTCGATGAT | 58 |
| CXCL3 | tcactgaactgcgctgccagtgcttgc  GTTGGTGCTCCCCTTGTTCAGTATCT | 58 |
| CXCL4 | ctgcgcctcacgccccgggctgctgt  GTACAGCGGGGCTTGCAGGTCCAAGCA | 60 |
| CXCL4L1 | cgcagccaggtcccgcctcacccg  ATGATTTTCTTGTACAGCAGGGCT | 60 |
| CXCL5 | gcagccagggcccatcgccagcgctg  CGTGCTCATTTCTCTTAATCAGTTTT | 60 |
| CXCL6 | tgctgctcctgctgacgccgccggggc  ACTGTCCAAAATTTTCTGGATGACTTT | 60 |
| CXCL7 | ccaaaggacaaactaagagaaact  AACAAATTAATCAGCAGATTCATCAC | 58 |
| CXCL8 | gacttccaagctggccgtggctctcttg  TGTTGGCGCAGTGTGGTCCACTCTCA | 60 |
| CXCL9 | agggtcgctgttcctgcatcagcaccaa  ACGTTGAGATTTTCGAACTTTCAGAAC | 60 |
| CXCL10 | taagtggcattcaaggagtacctctctc  AGGAGATCTTTTAGACCTTTCCTTGCT | 60 |
| CXCL11 | tagccttggctgtgatattgtgtgctacag  AAGCCTTGCTTGCTTCGATTTGGGA | 58 |
| β-actin | CACTCTTCCAGCCTTCCTTC  GTACAGGTCTTTGCGGATGT | 60 |
| Enhancer a | TAATGTTAAATTGAAGATTTAG  AGTTATACATGCCTGGTCTATA | 60 |
| Enhancer b | GGTACCGGCGCCCTGGG  GAGCGAGGCTGTCGGCGCTG | 60 |
| Enhancer c | TTACGATAATTACTGCTCTGGTGC  CGGTGGTCTCCACTGTACTATTG | 60 |
| Enhancer d | AGAATAATTGCATTCTGATACA  TGTACAACAAAATGCTATCTCTAA | 60 |
| Enhancer e | CTTGCCTATGTTACTTAACTTCTTGT  GATGGGCTTTGTGTACTGACCTCAT | 60 |
| ab for 3C | TTGACTGCTAAGATTATATTC  AGACTGCGTAAAATCCCGTCATT | 52 |
| ac for 3C | TTGACTGCTAAGATTATATTC  CCAATAGTACAGTGGAGACCA | 52 |
| ad for 3C | TTGACTGCTAAGATTATATTC  CTCTTTGGTTTTTATTTCTTTGGT | 54 |
| ae for 3C | TTGACTGCTAAGATTATATTC  atgttgggaatccccagggctTTc | 55 |
| bc for 3C | AACGTATGCAAGCAACAATTCT  CCAATAGTACAGTGGAGACCA | 55 |
| bd for 3C | AACGTATGCAAGCAACAATTCT  CTCTTTGGTTTTTATTTCTTTGGT | 55 |
| be for 3C | AACGTATGCAAGCAACAATTCT  atgttgggaatccccagggctTTc | 52 |
| cd for 3C | AAACAGCCAGAACCTGTTGCTGTTT  CTCTTTGGTTTTTATTTCTTTGGT | 52 |
| ce for 3C | AAACAGCCAGAACCTGTTGCTGTTT  atgttgggaatccccagggctTTc | 55 |
| de for 3C | atggccaattgcaaaatagaaa  atgttgggaatccccagggctTTc | 52 |
| positive control | TGGGCTCAAAGGTGAGAGAA  CACTGACAACTGTGTTCACTAGC | 58 |
| negative control | ATTTTGACCCTGAGGATGG  GCGCGCGACTTCATGCGTA | 60 |
| gDNA for a | ATACATAATATAGGTAACCG(TGG) |  |
| gDNA for b | TTTCTGTTAATTCTTTCTGA(AGG) |  |
| gDNA for c | GTGCCTACCCCAGCCGCGTC(CGG) |  |
| gDNA for d | TTTTCATTTTGAAGATGACG(TGG) |  |
| gDNA for e | AAAGATTAGGTTGAAAAATT(AGG) |  |
| Enhancer a | TAAGCACATCTTTCTGACCTACAGCGTTTTCCTATGTCTAAATGTGATCCTTAGATAGCAAAGCTATTCTTGATGCTTTGGTAACAAACATCCTTTTTATTCAGAAACAGAATATAATCTTAGCAGTCAATTAATGTTAAATTGAAGATTTAGAAAAAACTATATATAACACTTAGGAAAGTATAAAGTTTGATCAATATAGATATTCTGCTTTTATAATTTATACCATGTAGCATGCATATATTTAACGTAAATAAGTAATTTATAGTATGTCCTATTGAGAACCACGGTTACCTATATTATGTATTAATATTGAGTTGAGCAAGGTAACTCAGACAATTCCACTCCTTGTAGTATTTCATTGACAAGCCTCAGATTTGTCATTAATTCCTGTCTGGTTTAAAGATACCCTGATTATAGACCAGGCATGTATAACTTATTTATATATTTCTGTTAATTCTTTCTGAAGGCAATTTCTATGCTGGAGAGTCTTAGCTTGCCTACTATAAATAACACTGTGGTATCACAGAGGATTATGCAATATTGACCAGATAAAAATACCATGAAGATGTTGATATTGTACAAAAAGAACTCTAACTCTTTATATAGGAAGTCGTTCAATGTTGTCAGTTATGACTGTTTTTTAAAACAAAGAACTAACTGAGGTCAAGGGCTAGGAGAATATTCAGGAATGAGTTCACTAGAAACATGATGCCTTCCATAGTCTCCAAATAATCATATTGGAATTAGAAAGGAAGTAGCTGGCAGAGCTGTGCCTGTTGATAAAATCAATCCTTAATCACTTTTTCC |  |
| Enhancer b | GGTACCGGCGCCCTGGGGTCCCCGGGCCGGACGCGGCTGGGGTAGGCACCCAGCGCCGACAGCCTCGCTC |  |
| Enhancer c | GTTATGGTTTCCATGTACACAGGCGACTGGAGCCGTTGGTCAGAAATACTGGCATGTGCCCCCTAAAAATAAAATCAGGAAAACCCAGGGGTTAGTTGAAGGACTAGAAATTGGGATTATTGTTTTCACAATTAAGGTTTCCTTTACGATAATTACTGCTCTGGTGCCAGAGGATATTCCCAATGCCTGGCGTCCCCACCCTGGTTCTTCCTTCGTTCCAATGAATGTAGGTAAAACTGCCTTCATTTGAGGCCCAGTAGGACAAACAGCAACAGGTTCTGGCTGTTTTTAATCCAATAGTACAGTGGAGACCACCGCCCCACCCCACCCCCATTCCTAAAAGAGCATCCCAAGCTTAGAGGTCCCTGCCACACAGCACAGCTGTCATAGGCAGTAGCCACTTGGTTGCCAGGCTGGGGAAACTGCATTCGGAAAACTCTAGAGGCTGGAGGAGCAGGGCAGGAGAAGAGTGTTGTGCAATCAGCTTTCCCGAGCACCTACTCAGGGCACCCATTTTCTC |  |
| Enhancer d | cactgatagcagtccactgtaagagcaagcatatttttgcattctgcttcaaagccttccccaaggtagtgggagtctgcttgactagtatggaaaaagagtcccaagtagaggaagagagtttatgtccctgggagtaatcctcaaccactaggcaaggagaactgatggttacctgcaccagactccaggccttcaaggcaagcaattccagaaagcattctatgtgctccccacaaattccagaggaatggaggccctgtttctcatagcagcaactcagtgaggcatacatttatactggcttcccactcctccaggtttcactcctcttacctcttatgtttgtgtcttggataccctcgcaaatcaactacaaggacccaagtccttgtctgaggttctgattttaggagagtaaaaattatggcaATTCCAAAAGaataaaaaataaaaataaaaaatCTCCTGCCTACAAAAACACATTTTTTTCTGGGTGTTCTATAGATTATAAAAACATTTATTTTTCTATTTTGCAATTGGCCATTTTAAAGCTCTTTGGTTTTTATTTCTTTGGTCCTGTGTTGCATTCTCATTTCAGAAGTAGCCAGTTAGCCCAAAGCGTTTATTTACTTTAGTCTTAGTGTGGTAATGGTACCGTCCCCAATGTTCTGCCTGTCACTTTTCTTAGTTTAATGTTGTAGTAAACAAAGTTAAGACACCACCATAATTCTGAGGTATGTTTAACACTTGCTCAAGAGGTTAACTTCTTGTGTTTTTTTCCCAAATGACCTGATTCCCTCATAGTCTCTGTGGTTAGTTGTATTTGGAGGTATTTGGCTCTGAATTTAAACATTTAAATGTGGTTGGCCTTTGGAAAACACCAGACAGTGAGTCAGAGGCTTTTCATTCCTTTCTATGATTCTGAGCATTATGTTGAGAAACCTCCTACCCTCTCTAGGCAATTTCTCCATCTGTAATGGAGCCACCCAGCACTTTTCTGTATCCTGGATTAGAGTCAGTGTTAATAGCACTGGGAGACTCTTTGATACTTTAATCAGAAATGGGCCTAATTCCATTAAATTTTCTTGTGTTTATAATCTAAGcaggggtgcccaatccccgggctatcagtctgtcccctgttaggaactgggccacacagcaggaggtgagcagcaggcaagacagcattactgcttgagctccatctctgataaaattagattctcataggagcatgaaccctattgtgaactgcatgcgaggtttataggttgcgagctccttgtgagaatttaactaatgcctgatgatctgaggtggggcagtttcattcccgaaactactcccccaccctcaccatccttggaaaaattgtcttccacaaaacaggtccctggtgccaaaaaaaaggttgaggaccgctgATTTAAAGTACTTCTATTAGTTGTTATATTTTGTCGTGTCTGGAAAAATGTGTTAACTATAAGAGAAGAGAGAATGATCTTTTGTACTTTGATAGAATAATTGCATTCTGATACAGGAAAATAAAACTATTTCTAAGTGGGGGAAACAAAATCATTTCTTAAAACACTGAATAGACTATTAGCCCTTTACATGGCTTTCTGAGTCTCATTCTGGCCAAGTCAATACTATGTTGCCTCCTAGTTTTACAAGGGTACATTCCTTCCAATAAAACAATGTGTGAATCCACAGGACACTCCAAGAAAATGAGGAATGGCATTTATTGGTACCTTGTTAGAACACAAGCATAAACCTCTACTGCACTAAAGCTGTGTGTGAAAAGAGTCTGGGAGAGAAGGAAGGGGCGGTATGGCAGCAAATGATTCTATAGGGAtgcgtgggagtgtgtgtgtgtgtgtgttttctcagtctgtgtatgtgtgtTTTCTGGGTGTTGTTAAGAGGTAGTCTGTAATAAGAATATAACTCCACTTACTTTTCTATTAGAGATAGCATTTTGTTGTACAAATTCTCTTTGAAGTTCTTTGATAAATCTTTGGCTCATTCATTAGTATCTTAGGGTCAAGTGCTATAAACAGATTAATAACTTATTTTGTTTTCAGCACTGATATGGAAGGGGATTGGGCATGGGGCGTGAGGAATAATTTTCATTTTGAAGATGACGTGGATCTGCATGACAGTCTCTGGTCTTCCAGAGAGAAGTGACAAAGCTCTAGGTTCCAGAGATACAAGAATGAGGAGGAGGCAAAACAGCTTGGGAATGAAGGTGAAAGCTGACAAAAAGAAAAACTGTTTGACAGTTGTTGACTTTGCAATTTTTCCTGATAAACTCTTACAAAAGTATCTCCAGGTTAGTATGAAGATAAGAGAACATCCCTATCGATTTATCCCAGTTTTCCCAGTCAAGTCTGTGTCCCAAATGGAAAAAGAGAAAAAGAGACCAATTAAAAGGAACTAGAAAAGAAAAAAACTATATATTACTAAACACACATGCACTCGCACACACACACCAAGGAGTTCTTGAGCTCATTTTCCCTGTCTTTCATACTTACCAACAAAAATATTTAAAACATTAGGATTTATATCCTCTAA |  |
| Enhancer e | caccgtgcccgaccccatgttgctaaatttaatggtggtcaacttttggtattcatcttatttgactcatcagcatctgatgtgcttgggttctccctcccttgggaacatcttctatacttggcttccagaacacctcctggttttcctcgaaacttcactggctgctttctcagtccctttttccagttacttagaaactcactggcctttaaatgttgggaatccccagggctTTctctacctacccaatccaggatctaatcacccttcttactacttcctgttaccatcctagcaccactcaacccagcatcatctcttgctgtggataattccaatagcttcttatgtggtgtctctgcttctatctttgccctcctacatacagtctatcctcaagtaacggtgatcttgttctcatatagggtggttcatgtattcttctgctcaaaaccctgtaaaggcttccatctcattgggaggtagaatcccaaattcaccccctttcctcattccctatttggagccacactggcctttatgttcctgaaatctaccaggcatacctaccccaaggtctttataccttctgttccttctgcctagatcattcttcctaaacatatgcacagctctccctcatgtccttcaggttttcactcaaagattactatctcaagaagaccttccctaactatccaatttaaaactgcaatacccatctcacccccaacatacataagtactttcctattgtctttttttcacagcacttttcattatttaacatactatattatttgttactgtatatcaccccctactagaatgtcatttaatgagcatagaatttttatcggtacagctcaatattatgtagtcccagggcccagcattgtgccttacatatagcaggtaatcaacagtatttgtcaaatgaacgaaCTtcaggaaatggcactatcatctacctagacatataaataaaaaacctgggtgatccttcattccttcttttttccttatcccccacatccactccaccagaaatcccaaggctctactagctctacctccacgtagaacagcacgaatctttgcccataattccaatgacaccacaatcccttgcttggttattctgagatccattatattagttaccctgcttccagtgttgctcctctacaagccattccacatgtaacttccagagtaaattcttaaaaaaaatatgtcagatcatgtcactctcctgcttaaaactcttcaatcactccccgaggcactcagagtgaaatctaaactcgttaacagggcctaagaggctctgaataagctgacccctgcctgcatttcctacatcatcttccactgccctcttctatgctgactacactttagctacactggcttcttctctgctttttttccaccagtagcttctgcactttctattccttgtacctaaaatgatgtttacctgcctggctcaatatcatcattgcagagccaccttccctgtttacggggtctaaatcagaagcctccaattctaacctattcccatccagttttattttcttcataatctctaccataataagtctacactcacttgaatgtaagtccaagagaagaatgctttcttatctgtcttattcattactgtaccccaacgcctacctagaataacaacacacagtagTTATATGTGATTTTGCATATATACTTAAGTGAATTAAAgaagtagttattacccccttcttacatattaaaatacccacataggctcagcgattaacctgcttcggttacacaactggttaacaacagtcagcagcaggaGTTATCTAACGGAATTATATAGACTCTCAACTTACCACTTTCTTTGCGAATGGCTATACAATGCTTTAAGCATCTCAAAGCCTTGCCTATGTTACTTAACTTCTTGTACTTGTTTCCTACTCTTTATTTCCACTACCTTGATCCTTGGAGAGGCTTTCTCTACTGGTGACCTAAGCCACTGAGATTGCCATGTGTTAAAAAGCACTCATAAAATACATATATGTGAAAATTTAGGGGAGGAGGGAAGTGAGAAGGGCAATGAAAAAATAAGTAATTGTGTTAGCCACTGAACAAATCACAATCTGGTAGAAGCACAAACATGTAATTGCAGCAAATGCCTAGCAGAGATACCTGTTTAATGCCCGTCTGATAAATGAGTGTCTGAAGGAAGCACCTGTCAGATTAATATATCAAAAACCACTTTCGTTTTGTCGGTACTTGGTTCAAACACCCCTAATGTTCACAACTTACATGAGGTCAGTACACAAAGCCCATCACAATGAGGGCCTAATTTTTCAACCTAATCTTTTCCCGAAGCAAATTCTGACATCCGAGTCATGCCAGTCTCCGGTAAGTTTGAATCCTGGCTTAACTAAATATACTAAGCTCCCGAGTCTGTCCGTTGTGCTTGAGAAACCCCCCCCCCCCCTTTCCTGGCACAGTCCAGGGTACACAGCCTGCTCTCAGAAACGGACGTCAATCGAAAGCATTCTAACATACGATTCATCAAAGGTTACCGACTAATTCAAGTTTGGGATTTCTGAAAAGAGGCTGAGAAATCCGCGTGGAAGTTGGTGGTTGGGTGCGGCCCATTTCTCTCTCCACTTCCGTGCGGACGAAGAAAAATATCTTAAAACAAAATGAGGTTTTAAAGGTGTTTCAGCGGCAGCAGGGCAGGTAGAAAATCCAAAGGGGCAACAGATTTTCAAGAGGTGGGGAACGAACCCTAAGGCATGTCCCGTCCCCAACCCCTTCTCTCCTCTGGAGGGACCCCTGAACCTAACAGAAGAATAGGCGGCGAGCCTGGATCCCAGCCCCACTCCATGCTTTCCCGCACCGGCGTCTCAACCATCCACTATCCGGCCTCTAGCGTCTGCCGCGCCGCACTC |  |

Table S1. Sequences of primer, gDNA and enhancer are listed.
